# INFECTION PREVENTION CHALLENGES AND OPPORTUNITIES IN SMALL LONG-TERM CARE FACILITIES

**DOI:** 10.1093/geroni/igac059.031

**Published:** 2022-12-20

**Authors:** Carolyn Ham

**Affiliations:** Washington State Department of Health, Shoreline, Washington, United States

## Abstract

Small, individually owned long-term care facilities experienced unique challenges to accessing infection prevention knowledge and resources during the COVID-19 pandemic. Three data sets were analyzed: 1) Multi-state qualitative interviews with public health and regulatory staff in spring 2021; 2) Online survey of Washington state adult family home providers on infection control knowledge and practices in fall 2020; 3) Non-regulatory Infection Control Assessment and Response evaluations conducted between March 2020 and January 2021. Consistent findings across datasets were ongoing difficulty obtaining personal protective equipment (PPE), inability to implement isolation precautions and high vulnerability to staffing shortages. Small facilities showed strengths in consistency of leadership and engagement with public health outreach. Facility size is a key factor in infection prevention disparities for older adults living in residential care. Promising policy interventions are prioritization of small facilities for PPE distribution and increasing the availability of public health infection prevention expertise.

